# Hypoalbuminemia on Admission as an Independent Risk Factor for Acute Functional Decline after Infection

**DOI:** 10.3390/nu13010026

**Published:** 2020-12-23

**Authors:** Hidehiko Nakano, Hideki Hashimoto, Masaki Mochizuki, Hiromu Naraba, Yuji Takahashi, Tomohiro Sonoo, Kensuke Nakamura

**Affiliations:** Department of Emergency and Critical Care Medicine, Hitachi General Hospital, Ibaraki 317-0077, Japan; hidehashimoto-tky@umin.ac.jp (H.H.); kurakan72@gmail.com (M.M.); nrbhrm@gmail.com (H.N.); yuji.mail@icloud.com (Y.T.); sonopy77@gmail.com (T.S.); mamashockpapashock@yahoo.co.jp (K.N.)

**Keywords:** albumin, nutrition, frailty, sarcopenia, post-intensive care syndrome, post-acute care syndrome

## Abstract

The risk of acute functional decline increases with age, and concepts including frailty and post-acute care syndrome have been proposed; however, the effects of the nutritional status currently remain unclear. Patients admitted to the emergency department of Hitachi General Hospital for infectious diseases between April 2018 and May 2019 were included. To identify risk factors for functional decline at discharge, defined as Barthel Index <60, we investigated basic characteristics, such as age, sex, disease severity, the pre-morbid care status, and cognitive impairment, as well as laboratory data on admission, including albumin as a nutritional assessment indicator. In total, 460 surviving patients out of 610 hospitalized for infection were analyzed. In a multivariable logistic regression analysis, factors independently associated with Barthel Index <60 at discharge were age (adjusted OR 1.03, 95%CI 1.01–1.06, *p* = 0.022), serum albumin (adjusted OR: 0.63, 95%CI: 0.41–0.99, *p* = 0.043), and the need for care prior to admission (adjusted OR: 5.92, 95%CI: 3.15–11.15, *p* < 0.001). Hypoalbuminemia on admission in addition to age and the need for care prior to admission were identified as risk factors for functional decline in patients hospitalized for infection. Functional decline did not correlate with the severity of illness.

## 1. Introduction

Acute functional decline is associated with poor outcomes, and is referred to as post-intensive-care syndrome (PICS) [1] or intensive care unit (ICU)-acquired weakness (AW) among critically ill patients [2]. Aging is a major risk factor for ICU-AW [3], and the risk of functional decline is higher among elderly patients, even if they are not admitted to the ICU. Therefore, functional decline needs to be considered in elderly patients regardless of ICU admission or disease severity as post-acute care syndrome (PACS) [4]. Age-related post-disease functional decline is a contributing factor to frailty, which is characterized by a decreased ability to recover from stress, and aging has been identified as the main risk factor for acute functional decline [4,5].

Sepsis is one of the most common causes of functional decline, both as ICU-AW and PICS [6]. Aging is also a major risk factor for ICU-AW in patients with sepsis [7], together with other factors, such as gender, disease severity, immobilization, hyperglycemia, and the use of steroids and neuromuscular blocking agents [8]. Therefore, the development of strategies to prevent PICS and ICU-AW is urgently needed.

Limited information is currently available on the nutritional status as a risk factor for PICS and ICU-AW [9]. Nutrition is an important factor in the chronic phases of frailty and sarcopenia [10,11,12]. Previous studies showed that protein intake was directly related to skeletal muscle mass in the elderly [13] and dose-dependently decreased the risk of frailty [14]. Reduced cognitive function and social activity have been identified as risk factors for functional decline; however, the effects of the nutritional status have not yet been examined in detail [15]. Therefore, it currently remains unclear whether a direct relationship exists between the nutritional status and physical function, not muscle mass.

We hypothesized that the nutritional status on admission, in addition to age, is a risk factor for functional decline. In the present study, we examined age, disease severity, and laboratory data on admission, including albumin, in patients admitted to our emergency department with infectious diseases in order to identify risk factors for functional decline at discharge.

## 2. Materials and Methods

### 2.1. Study Design and Participants

A single-center retrospective observational study was conducted at a critical care center with 18 beds in a tertiary care hospital in East Japan. Patients admitted to our department between April 2018 and May 2019 were enrolled. We selected patients admitted with infectious disease based on the diagnosis in clinical records and those older than 15 years.

### 2.2. Data Collection

Data were collected through a review of clinical records. As basic characteristics, information was collected on age, sex, disease severity, length of hospitalization, ICU admission, and adjunctive therapy (mechanical ventilation and renal replacement therapy). Based on their relationship with physical function at discharge [15,16], information on baseline physical function, namely, the need for care prior to admission and cognitive impairment, was also extracted. The sequential organ failure assessment (SOFA) and acute physiology and chronic health evaluation (APACHE) II were used to evaluate disease severity. SOFA and APACHE II scores were calculated during the first 24 h of admission. The use of long-term care insurance was investigated in the baseline physical function assessment. Long-term care insurance services are provided in Japan for individuals aged 65 years or older who need care or support as well as for those aged 40–64 years who develop aging-related diseases and, thus, also require care or support. Long-term care insurance was classified into seven grades based on the care needs. A previous report showed that if the patient did not use long-term care insurance, the Barthel Index was considered to be almost 100, and the Barthel Index was declining as the grades of long-term care insurance went up [17]. Assessments of long-term care insurance use and cognitive impairment were based on an interview with patients or their surrogates on admission. Serum albumin levels on admission were used to evaluate the nutritional status. We also calculated the controlling nutritional status (CONUT) score, which is based on three variables: serum albumin levels, the lymphocyte count, and total cholesterol level, and rated on a scale of 0–12 [18]. We used the Barthel Index (BI) to evaluate the physical functional status at discharge [19]. Since BI < 60 was previously shown to indicate functional dependence, we set the cut-off value for poor physical function to BI < 60 [20]. BI at discharge was evaluated by nurses. Patients who died during hospitalization were excluded from the BI evaluation.

### 2.3. Outcome Evaluation

Patients were stratified according to age into four groups (16–64 years, 65–74 years, 75–84 years, and 85 years and older) and the overview of patients included in each age group was reviewed to assess the impact of age on the premorbid status, disease severity, and physical function after acute illness. We also compared patients that died and those that survived. Patients who were discharged alive were then divided into two groups (BI < 60 and BI ≥ 60 at discharge) and their basic characteristics and laboratory data on admission were compared to assess risk factors for acute functional decline. To adjust for the impact of age on acute functional decline, we also analyzed data on patients younger than 75 years and those 75 years or older. In the present study, elderly was defined as 75 years or older according to the Japan Geriatrics Society [21]. As a sensitivity analysis, we also checked the comparison of BI < 60 and BI ≥ 60 in non-infected patients during the same period.

To identify risk factors for functional decline at discharge, a multivariable logistic regression analysis of BI < 60 at discharge in surviving patients was performed. Age, sex, disease severity, the need for care prior to admission, cognitive impairment, and albumin levels were selected as explanatory variables. We also performed an exploratory univariable analysis using laboratory data on admission and selected items with *p* < 0.10 in the univariable analysis as explanatory variables for the multivariable analysis.

### 2.4. Statistical Analysis

The normality of the distribution of each parameter was assessed using the Shapiro–Wilk test. Differences between two groups were evaluated using the Student’s t-test for normally distributed continuous variables, the Mann–Whitney U test for non-parametric continuous variables, and the chi-squared test or Fisher’s exact test where appropriate for categorical variables. In multiple comparison, we used Kruskal-Wallis test for non-parametric variables, and chi-square test for categorical variables. A P-value < 0.05 was considered to indicate significance. In the multivariable logistic analysis, variance inflation factors were calculated for all explanatory variables to confirm multicollinearity. Statistical analyses were performed using R (version 3.6.1., R Foundation for Statistical Computing, Vienna, Austria).

## 3. Results

### 3.1. Descriptives

**Figure 1:**
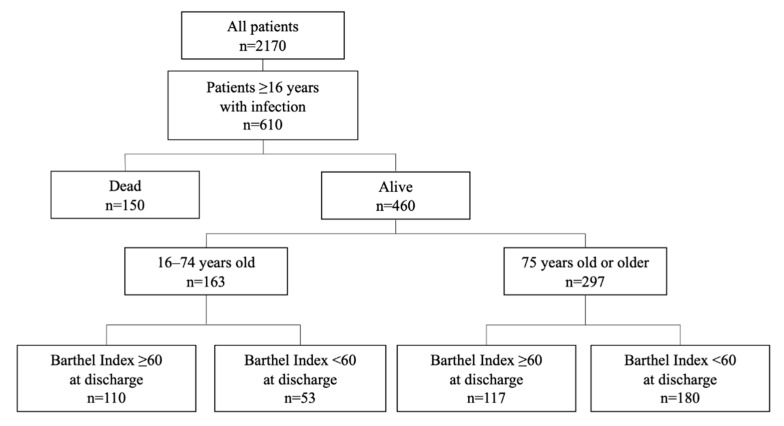
Patients included in the present study.

Among 2170 patients hospitalized in our department, 610 older than 15 years had infections and, thus, were included in the present study (Figure 1).

Table 1 shows an overview of patients in each age group. The median age, SOFA, and APACHE II of patients were 79.0, 4.0, and 14.0, respectively. The rate of surviving patients at discharge was 75.4% among all patients, and did not significantly differ between the age groups. On the other hand, the rates of ICU admission and ventilator use decreased with age. BI at discharge was lower in elderly patients (100.0 vs. 67.5 vs. 45.0 vs. 30.0, *p* < 0.001) and the rate of patients with BI < 60 at discharge increased with age (20.5% vs. 45.0% vs. 57.7% vs. 64.2%, *p* < 0.001).

Table 2 shows the characteristics of patients that died and those that survived. The patients died were older, more severely ill, and had more ICU admissions. There were no significant differences in length of hospital stay. The patients died had much lower albumin and higher CONUT score.

### 3.2. Acute Functional Decline and Basic Characteristics

The basic characteristics of 460 patients who were discharged alive are shown in Table 3. In the younger and older groups, patients with BI < 60 at discharge had a longer length of hospitalization and greater need for care prior to admission. In the younger group, patients with BI < 60 at discharge were older (62.0 (49.5–68.0) vs. 69.0 (61.0–71.0), *p* < 0.001) and had more severe illness (4.0 (3.0–7.0) vs. 6.0 (4.0–8.0), *p* = 0.017 for SOFA, and 12.0 (7.0–17.0) vs. 16.0 (12.0–20.0), *p* = 0.003 for APACHE II). In the older group, the rate of cognitive impairment was higher among patients with BI < 60 at discharge (18.7% vs. 38.3%, *p* < 0.001). As a sensitivity analysis, of 1400 patients admitted with non-infectious diseases and discharged alive, we analyzed 968 patients with recorded BI at discharge and found significant differences in age (*p* < 0.001), male sex (*p* = 0.013), APACHE II (0.036), and albumin (*p* < 0.001) (Table S1).

### 3.3. Acute Functional Decline and Laboratory Data on Admission

Table 4 shows comparisons of laboratory data on admission. Patients with BI <60 at discharge had lower lymphocyte counts and hemoglobin and serum albumin levels as well as higher blood urea nitrogen levels and CONUT scores. Serum albumin was the only factor that significantly differed in both the younger and older groups (3.12 ± 0.78 vs. 2.73 ± 0.81, *p* = 0.004 in the younger group and 3.08 ± 0.66 vs. 2.87 ± 0.66, *p* = 0.006 in the older group).

### 3.4. Risk Factors for Acute Functional Decline

The results of a multivariable logistic regression analysis of BI <60 at discharge are shown in Table 5. We used age, sex, disease severity, long-term care insurance use, cognitive impairment, and albumin as explanatory variables based on the results of previous studies. For severity, we used APACHE II, which was more differential in univariable analysis rather than SOFA. We also added hemoglobin (*p* < 0.001), platelet (*p* = 0.052), and blood urea nitrogen (*p* = 0.003), of which *p* value was <0.10 in the univariable analysis of laboratory data on admission, as explanatory variables. Because albumin showed a stronger difference (*p* < 0.001), CONUT score (*p* = 0.001) was not included in the multivariable analysis due to multicollinearity issues. Factors independently associated with BI <60 at discharge were age (adjusted OR 1.03, 95%CI 1.01–1.06, *p* = 0.022), serum albumin (adjusted OR: 0.63, 95%CI: 0.41–0.99, *p* = 0.043), and the need for care prior to admission (adjusted OR: 5.92, 95%CI: 3.15–11.15, *p* < 0.001). APACHE II scores were not independently associated with BI < 60 at discharge. We performed multiple regression analysis with the Barthel Index as the continuous variable and also with the same explanatory variables. This analysis also showed an independent association for albumin (Table S2).

## 4. Discussion

Among patients admitted to our hospital with infectious diseases, no significant differences were observed in survival or the severity of illness between the younger and older groups, whereas BI at discharge was worse in the older group. In addition to an older age and poor pre-morbid care status, hypoalbuminemia was independently associated with a low BI at discharge.

As expected, age was identified as a major risk factor for poor physical function at discharge. The present results are consistent with the concept of PACS because acute functional decline in elderly patients is independent of disease severity [4]. This is also indicative of frailty, which is characterized by a decreased ability to recover from stress, particularly in the elderly [10,11].

In the present study, serum albumin level on admission was an independent risk factor for acute functional decline at discharge. Albumin is one of the indicators of nutritional status and is included in nutritional assessment scores, such as CONUT score [18]. It was reported that serum albumin level on admission is associated with nosocomial infection or mortality during admission [22,23,24]. While albumin can be a good marker of nutritional status in a clinically stable elderly people in the community [25], serum albumin level is also lowered by inflammation, liver disfunction, and kidney disfunction [26,27,28] and is not a good marker of nutrition during acute phase; however, because of its long half-life of 14–20 days, serum albumin levels on admission immediately after the onset of the disease are less susceptible to changes due to inflammation and may reflect baseline status [28,29].

Although its reliability as a nutritional indicator remains controversial, previous studies reported that low albumin is correlated with muscle mass loss. A cross sectional study in healthy older adults found that low albumin was correlated with low muscle mass [30], and another study showed that even if the muscle mass in the baseline is the same, the elderly with lower albumin had greater muscle mass loss after 5 years [31]. However, the relationship between nutritional status or albumin and muscle mass loss during the acute phase has not yet been studied in detail [32], and furthermore, muscle mass and physical function are not always directly linked [33,34]. Nevertheless, the loss of muscle volume during acute phase may be associated with delays in recovery and mobilization [35], leading to adverse health outcomes and a poor quality of life [36,37]. A smaller muscle volume has also been associated with reductions in strength and mobility after critical care [38]. Thus, the result showing that albumin levels on admission are associated with functional decline at discharge is of importance.

Limited information is currently available on nutritional indicators and risk factors for functional decline at discharge [15]. The only study that included laboratory data was by Wu et al. [39], in which albumin was examined as a risk factor, similar to the present study. The present results support the importance of assessing albumin levels on admission in patients with infections, regardless of severity, in order to identify those at an increased risk of functional decline at discharge. The early recognition of malnutrition and timely initiation of nutritional care may improve quality of life while reducing healthcare costs [40]. The effectiveness of acute-phase nutritional therapy after disease currently remains unclear. Future studies are needed to evaluate and validate treatment methods, such as acute-phase nutritional therapy and rehabilitation, in the acute phase of illness.

There are several limitations that need to be addressed. There may have been other confounding factors that we were unable to analyze due to the retrospective nature of this observational study. Furthermore, we did not obtain detailed information on pre-disease physical function. However, we added the use of long-term care insurance prior to admission to the multivariable analysis and attempted to adjust for the pre-disease functional status. In addition, albumin levels are affected by other factors besides nutrition, such as chronic diseases and inflammation; however, this was not evaluated in the present study. Furthermore, rapid turnover proteins, such as prealbumin, need to be considered. Another limitation of the present study is the use of nutrition therapy and rehabilitation methods during hospitalization. Moreover, we did not investigate long-term outcomes after discharge. Therefore, further studies are needed to develop appropriate intervention methods in order to achieve better long-term outcomes.

## 5. Conclusions

Hypoalbuminemia on admission in addition to age and the need for care prior to admission were identified as risk factors for acute functional decline in patients hospitalized for infection. Acute functional decline did not correlate with the severity of illness.

## Figures and Tables

**Table 1:** Overview of patients included in each age group.

	Overall	-64	65–74	75–84	85-	*p*
*n*	610	101	107	216	186	
Age (years)	80.0 (70.0–86.0)	56.0 (46.0–60.0)	70.0 (68.0–72.5)	80.0 (78.0–82.0)	89.0 (87.0–92.0)	<0.001 *
Male	383 (62.8)	62 (61.4)	76 (71.0)	133 (61.6)	112 (60.2)	0.277
Survival discharge	460 (75.4)	83 (82.2)	80 (74.8)	163 (75.5)	134 (72.0)	0.301
SOFA score	5.0 (3.0–8.0)	6.0 (3.0–9.0)	5.0 (3.0–9.0)	5.0 (3.0–8.0)	4.0 (2.0–8.00)	0.042 *
APACHE II score	15.0 (11.0–21.0)	14.0 (11.0–20.8)	15.00 (10.0–21.0)	16.0 (12.0–21.5)	14.0 (11.0–19.0)	0.154
Need for care prior to admission †	211 (43.5)	3 (3.9)	22 (27.8)	72 (41.1)	114 (73.5)	<0.001 *
Cognitive impairment	145 (28.5)	19 (24.1)	11 (13.8)	59 (32.1)	56 (33.9)	0.005 *
Length of hospitalization (days)	9.0 (5.0–17.0)	9.0 (3.0–15.0)	9.0 (5.0–15.0)	10.0 (6.0–18.0)	9.0 (5.3–17.8)	0.268
ICU admission	187 (30.7)	37 (36.6)	42 (39.3)	76 (35.2)	32 (17.2)	<0.001 *
Mechanical ventilation	153 (25.1)	27 (26.7)	35 (32.7)	66 (30.6)	25 (13.4)	<0.001 *
Renal replacement therapy	105 (17.2)	24 (23.8)	25 (23.4)	34 (15.7)	22 (11.8)	0.019 *
Barthel Index ‡	55.0 (10.0–100.0)	100.0 (67.5–100.0)	67.5 (15.0–100.0)	45.0 (0.0–85.0)	30.0 (0.0–70.0)	<0.001 *
Barthel Index < 60 ‡	233 (50.7)	17 (20.5)	36 (45.0)	94 (57.7)	86 (64.2)	<0.001 *

*: *p* value < 0.05, †: Use of long-term care insurance, ‡: value of 460 surviving patients at discharge. Values are shown as median (IQR) or n (%). IQR, interquartile range; SOFA, sequential organ failure assessment; APACHE, acute physiology and chronic health evaluation; ICU, intensive care unit.

**Table 2:** Overview of dead and surviving discharged patients.

	Died	Survived	*p*
*n*	150	460	
Age (years)	81.0 (71.2–88.8)	79.0 (69.0–85.2)	0.011 *
Male	107 (71.3)	276 (60.0)	0.017 *
SOFA score	8.0 (5.0–11.0)	4.0 (2.0–7.0)	<0.001 *
APACHE II score	21.0 (14.0–25.0)	14.0 (10.0–18.2)	<0.001 *
Need for care prior to admission †	60 (52.2)	151 (40.8)	0.041 *
Cognitive impairment	43 (35.8)	102 (26.3)	0.056
Length of hospitalization (days)	10.5 (4.0–20.0)	9.0 (5.0–16.8)	0.574
ICU admission	77 (51.3)	110 (23.9)	<0.001 *
Mechanical ventilation	71 (47.3)	82 (17.8)	<0.001 *
Renal replacement therapy	51 (34.0)	54 (11.7)	<0.001 *
ALB (g/dL)	2.5 (0.6)	3.0 (0.7)	<0.001 *
CONUT score	8.0 (6.0–10.0)	6.0 (4.0–9.0)	<0.001 *

*: *p* value < 0.05, † Use of long-term care insurance. Values are shown as median (IQR) or n (%). IQR, interquartile range; SOFA, sequential organ failure assessment; APACHE, acute physiology and chronic health evaluation; ICU, intensive care unit. ALB, albumin; CONUT, controlling nutritional status.

**Table 3:** Baseline characteristics of surviving patients stratified by the Barthel Index at discharge.

	Overall			Patients Younger Than 75 Years			Patients Aged 75 Years or Older		
	Barthel Index ≥ 60	Barthel Index < 60	*p*	Barthel Index ≥ 60	Barthel Index < 60	*p*	Barthel Index ≥ 60	Barthel Index < 60	*p*
*n*	227	233		110	53		117	180	
Age (years)	75.0 (62.5–83.0)	81.0 (75.0–87.0)	<0.001 *	62.0 (49.5–68.0)	69.0 (61.0–71.0)	<0.001 *	83.0 (78.0–87.0)	84.0 (80.0–89.0)	0.083
Male	139 (61.2)	137 (58.8)	0.662	72 (65.5)	32 (60.4)	0.647	67 (57.3)	105 (58.3)	0.951
SOFA score	4.0 (2.0–7.0)	4.0 (3.0–7.0)	0.189	4.0 (3.0–7.0)	6.0 (4.0–8.0)	0.017 *	4.0 (2.0–6.3)	4.0 (2.0–7.0)	0.664
APACHE II score	12.0 (9.0–18.0)	15.0 (11.0–19.0)	<0.001 *	12.0 (7.0–17.0)	16.0 (12.0–20.0)	0.003 *	13.0 (10.0–18.0)	15.0 (11.0–19.0)	0.065
Need for care prior to admission †	38 (19.9)	113 (63.1)	<0.001 *	1 (1.1)	17 (44.7)	<0.001 *	37 (35.9)	96 (68.1)	<0.001 *
Cognitive impairment	35 (17.8)	67 (35.1)	<0.001 *	15 (16.7)	8 (21.6)	0.685	20 (18.7)	59 (38.3)	0.001 *
Length of hospitalization (days)	7.0 (4.0–12.0)	11.5 (7.0–20.0)	<0.001 *	6.0 (3.0–12.8)	12.0 (8.0–18.0)	<0.001 *	7.0 (4.0–11.0)	11.0 (7.0–21.0)	<0.001 *
ICU admission	53 (23.3)	57 (24.5)	0.864	29 (26.4)	21 (39.6)	0.124	24 (20.5)	36 (20.0)	1
Mechanical ventilation	36 (15.9)	46 (19.7)	0.334	20 (18.2)	15 (28.3)	0.204	16 (13.7)	31 (17.2)	0.512
Renal replacement therapy	27 (11.9)	27 (11.6)	1	16 (14.5)	14 (26.4)	0.106	11 (9.4)	13 (7.2)	0.649

*: *p* value < 0.05, † Use of long-term care insurance. Values are shown as median (IQR) or n (%). IQR, interquartile range; SOFA, sequential organ failure assessment; APACHE, acute physiology and chronic health evaluation; ICU, intensive care unit.

**Table 4:** Laboratory data on admission of surviving patients stratified by the Barthel Index at discharge.

	Overall			Patients Younger Than 75 Years			Patients Aged 75 Years or Older		
	Barthel Index ≥ 60	Barthel Index < 60	*p*	Barthel Index ≥ 60	Barthel Index < 60	*p*	Barthel Index ≥ 60	Barthel Index < 60	*p*
*n*	227	233		110	53		117	180	
WBC (*10^2/µL)	96.0 (69.0–137.5)	98.0 (69.0–139.0)	0.956	94.0 (67.0–132.8)	100.0 (88.0–186.0)	0.012 *	103.0 (74.0–141.0)	92.0 (64.8–131.0)	0.144
Lymphocytes (/µL)	932 (626–1355)	750 (504–1353)	0.019 *	959 (720–1276)	1248 (693–1764)	0.159	900 (598–1458)	724 (478–1168)	0.008 *
HGB (g/dL)	12.40 ± 2.37	11.50 ± 2.65	<0.001 *	12.89 ± 2.52	10.79 ± 2.97	<0.001 *	11.96 ± 2.14	11.71 ± 2.52	0.383
PLT (*10^4/µL)	18.0 (12.6–23.7)	18.7 (13.0–25.6)	0.313	18.5 (12.5–24.4)	17.6 (12.6–29.4)	0.921	17.4 (13.9–23.1)	18.8 (13.3–25.0)	0.22
ALB (g/dL)	3.10 ± 0.72	2.84 ± 0.70	<0.001 *	3.12 ± 0.78	2.73 ± 0.81	0.004 *	3.08 ± 0.66	2.87 ± 0.66	0.006 *
TC (mg/dL)	141 (111–170)	139 (109–167)	0.677	137 (106–162)	135 (110–163)	0.975	145 (113–179)	143 (110–168)	0.289
T-Bil (mg/dL)	0.8 (0.6–1.2)	0.8 (0.5–1.2)	0.225	0.7 (0.5–1.1)	0.6 (0.4–1.1)	0.19	0.9 (0.6–1.3)	0.8 (0.6–1.2)	0.131
BUN (mg/dL)	21.0 (15.0–31.8)	25.1 (17.9–44.3)	0.001 *	18.5 (13.4–30.8)	27.9 (15.8–58.9)	0.004 *	22.9 (16.7–33.3)	24.8 (18.4–38.5)	0.173
CRE (mg/dL)	1.04 (0.77, 1.71)	1.03 (0.75, 1.79)	0.703	0.97 (0.73, 1.50)	1.05 (0.56, 4.08)	0.574	1.10 (0.83, 1.79)	1.02 (0.76, 1.67)	0.174
CRP (mg/dL)	7.32 (1.43–16.84)	7.18 (2.32–15.59)	0.952	6.46 (1.21–18.49)	11.40 (5.03–21.82)	0.125	7.92 (1.79–15.80)	6.83 (2.01–12.51)	0.562
PCT (ng/mL)	0.49 (0.13–5.42)	0.92 (0.21–6.57)	0.401	0.48 (0.12–6.31)	0.73 (0.19–4.70)	0.865	0.51 (0.15–4.65)	1.00 (0.21–6.93)	0.394
PT (sec)	1.10 (1.00–1.20)	1.10 (1.00–1.20)	0.73	1.10 (1.00–1.20)	1.10 (1.00–1.22)	0.371	1.10 (1.00–1.20)	1.10 (1.00–1.20)	0.726
CONUT score	6.0 (3.0–9.0)	7.0 (5.0–9.0)	0.001 *	6.0 (3.0–9.0)	8.0 (6.0–10.0)	0.127	5.0 (3.5–8.0)	7.0 (5.0–9.0)	0.003 *

*: *p* value < 0.05. Values are shown as mean ± SD or median (IQR). SD, standard deviation; IQR; interquartile range; WBC, White blood cell; HBG, hemoglobin; PLT, platelet; ALB, albumin; TC, total cholesterol; T-Bil, total bilirubin; BUN, blood urea nitrogen; CRE, creatinine; CRP, C-reactive protein; PCT, procalcitonin; PT, prothrombin time; CONUT, controlling nutritional status.

**Table 5:** Results of a logistic regression analysis of surviving patients with a Barthel Index < 60 at discharge.

Category	Crude OR	*p* Value	Adjusted OR	*p*
Age	1.01 (1.01–1.01)	<0.001 *	1.03 (1.00–1.06)	0.022 *
Sex	0.97 (0.89–1.07)	0.595	1.04 (0.60–1.81)	0.893
SOFA	1.01 (0.99–1.02)	0.267		
APACHE II	1.01 (1.00–1.01)	0.12	1.03 (0.99–1.06)	0.174
Length of hospitalization	1.00 (1.00–1.00)	0.004 *	1.00 (1.00–1.01)	0.296
Need for care prior to admission †	1.56 (1.42–1.72)	<0.001 *	5.92 (3.15–11.15)	<0.001 *
Cognitive impairment	1.25 (1.12–1.40)	<0.001 *	1.47 (0.79–2.73)	0.225
Lymphocytes ‡	1.00 (1.00–1.00)	0.145		
HGB ‡	0.97 (0.95–0.98)	<0.001 *	1.04 (0.92–1.17)	0.57
PLT ‡	1.00 (1.00–1.01)	0.052	1.02 (0.99–1.05)	0.144
ALB ‡	0.88 (0.83–0.94)	<0.001 *	0.63 (0.41–0.99)	0.043 *
BUN ‡	1.00 (1.00–1.00)	0.003 *	1.01 (1.00–1.02)	0.088
CONUT	1.03 (1.01–1.04)	0.001 *		

*: *p* value < 0.05, †: Use of long-term care insurance, ‡: laboratory data on admission. In consideration of multicollinearity, we adopted APACHE II for severity in the analysis and excluded SOFA. We also used albumin rather than CONUT score. OR, odds ratio; SOFA, sequential organ failure assessment; APACHE, acute physiology and chronic health evaluation; HGB, hemoglobin; PLT, platelet; ALB, albumin; BUN, blood urea nitrogen; CONUT, controlling nutritional status.

## Data Availability

The data presented in this study are available on request from the corresponding author. The data are not publicly available due to restrictions from the Ethics Committee.

## Supplementary Materials

The following are available online at https://www.mdpi.com/2072-6643/13/1/26/s1, Table S1: Comparison of non-infected surviving patients stratified by the Barthel Index at discharge., Table S2: Results of a multiple regression analysis of surviving patients with Barthel Index at discharge.
