# Supplementary material for: Hypoalbuminemia on Admission as an Independent Risk Factor for Acute Functional Decline after Infection
Source: Nutrients. 2020 Dec 23;13(1):26. doi: 10.3390/nu13010026 (PMC7823478; doi:10.3390/nu13010026)
Supplement: Supplementary file 1 [file nutrients-13-00026-s001.pdf]

Supplementary materials

Table S1. Comparison of non-infected surviving patients stratified by the Barthel Index at discharge.

	Barthel Index <60	Barthel Index ≥60	p
n	359	609	
**Age (years)**	80.0 [70.0–87.0]	68.0 [53.0–79.0]	<0.001*
**Male**	193 (53.8)	378 (62.1)	0.013*
**SOFA score**	3.0 [2.0–5.0]	4.0 [1.0–6.0]	0.749
**APACHE II score**	13.0 [10.0–18.0]	12.0 [8.0–18.0]	0.036*
**Length of hospitalization (days)**	3.0 [2.0–9.0]	2.0 [2.0–6.0]	<0.001*
**ICU admission**	48 (13.4)	71 (11.7)	0.495
**Mechanical ventilation**	29(8.1)	43(7.1)	0.649
**Renal replacement therapy**	21 (5.8)	28 (4.6)	0.48
**ALB (g/dl)**	3.5 (0.7)	3.8 (0.7)	<0.001*

*: p value < 0.05. Values are shown as median [IQR] or n(%). IQR, interquartile range; SOFA, sequential organ failure assessment; APACHE, acute physiology and chronic health evaluation; ICU, intensive care unit; ALB, albumin.

Table S2. Results of a multiple regression analysis of surviving patients with Barthel Index at discharge.

	β	p
Age	−0.171	0.004*
Sex	0.009	0.854
APACHE II	−0.088	0.089
Length of hospitalization	−0.021	0.65
Need for care prior to admission†	−0.375	<0.001*
Cognitive impairment	−0.111	0.028*
HGB‡	−0.036	0.538
PLT‡	−0.050	0.408
ALB‡	0.115	0.048*
BUN‡	−0.099	0.046*
R^2	0.291	<0.001*

*: *p* value < 0.05, †: Use of long-term care insurance, ‡: laboratory data on admission. β, standardized partial regression coefficient; APACHE, acute physiology and chronic health evaluation; HGB, hemoglobin; PLT, platelet; ALB, albumin; BUN, blood urea nitrogen; R^2, coefficient of determination.
